# Proton versus photon radiation therapy: A clinical review

**DOI:** 10.3389/fonc.2023.1133909

**Published:** 2023-03-29

**Authors:** Zhe Chen, Michael M. Dominello, Michael C. Joiner, Jay W. Burmeister

**Affiliations:** ^1^ School of Medicine, Wayne State University, Detroit, MI, United States; ^2^ Karmanos Cancer Institute, Department of Oncology, Wayne State University School of Medicine, Detroit, MI, United States

**Keywords:** proton radiation therapy, proton radiotherapy, proton therapy clinical trials, proton therapy non-randomized study, proton therapy randomized study

## Abstract

While proton radiation therapy offers substantially better dose distribution characteristics than photon radiation therapy in certain clinical applications, data demonstrating a quantifiable clinical advantage is still needed for many treatment sites. Unfortunately, the number of patients treated with proton radiation therapy is still comparatively small, in some part due to the lack of evidence of clear benefits over lower-cost photon-based treatments. This review is designed to present the comparative clinical outcomes between proton and photon therapies, and to provide an overview of the current state of knowledge regarding the effectiveness of proton radiation therapy.

## 1 Introduction

Radiation therapy is a primary method of cancer treatment, is used to treat approximately 50% of all cancer patients (1), and is a component of the treatment of 29% of cancer survivors in the United States (2). Advancements in photon radiation therapy techniques have steadily improved dose conformity around tumors, however, the high dose in adjacent normal tissues still limits dose escalation or even the delivery of necessary curative doses for certain types of cancers. Proton radiation therapy offers the potential for substantial improvements in the dose distribution in tumor and normal tissues, and may provide a clinical benefit for certain types of tumors, especially pediatric tumors and tumors located in anatomically challenging areas (3, 4).

As of October 2022, worldwide there were 118 proton radiation therapy centers in operation (42 in the United States) (5); 34 under construction (6); and 32 in the planning stage (7). However, as of December 2021 only an estimated 279,455 patients have been treated with proton radiation therapy worldwide (8). For comparison, IMV Medical Information Division estimates there were a total of 1.06 million radiation therapy patients in 2020 in the United States alone (9). A major hurdle for the use of proton radiation therapy is high treatment cost and lack of evidence of increased efficacy of proton radiation therapy over lower-cost photon-based treatments (10, 11). Therefore, randomized controlled comparative effectiveness trials are needed to qualify and quantify the potential superiority of proton radiation therapy in a given clinical scenario.

The aim of this review is to detail the comparative clinical outcomes between proton and photon therapies from existing and ongoing comparative clinical research literature and clinical trial protocols, and to provide an overview of the effectiveness of proton radiation therapy.

## 2 Methods and materials

The resources used in the compilation of research articles for this review included PubMed, ScienceDirect, Scopus, and Google Scholar databases. The Keywords or MeSH terms included “proton therapy,” “proton radiation therapy,” “proton beam therapy,” and “charged particle radiation therapy.” Filter criteria were as follows:

Inclusion criteria:

1. Comparative articles between external beam photon and proton radiation therapy (additionally, electron beam as part of a conventional photon-electron regimen)
2. Clinical research focused on radiation treatment-related clinical outcomes, which contained results for toxic effects, quality of life, local control, local recurrence, local failure, secondary malignancies, and survival rates
3. Published or accepted by 2021
4. In English

Exclusion criteria:

- Abstract only
- Treatment planning or dosimetric comparison articles
- Estimated or calculated clinical outcomes
- Cancer-related clinical outcomes
- Patient cohort less than five

In total, 63 interventional comparative articles meeting these criteria returned and were incorporated into this review, including 6 randomized studies (1 prostate, 3 lung, 1 esophagus, 1 adult central nervous system cancers) and 57 non-randomized reports (8 prostate, 3 breast, 9 lung, 8 esophagus, 7 head and neck, as well as 4 adult and 18 pediatric central nervous system cancers).

The clinical trial data for this review was collected from the U.S. National Library of Medicine ClinicalTrials.gov. Using headings including the same terms previously listed and selecting “Interventional Studies (Clinical Trials)” for study type, 196 clinical trials were identified. Among these, 36 comparative clinical trials aimed at evaluating clinical outcomes between external beam photon and proton radiation therapy were identified: 28 protocols recruiting, 2 completed, 3 not yet recruiting, 1 withdrawn, 1 active but not recruiting, 1 terminated.

## 3 Comparative clinical outcomes of notable cancer sites

In the following sections, comparative clinical outcomes between proton and photon therapies will be summarized. Additional information for selected non-randomized studies is shown in **Tables 1**–**7**, randomized studies in **Table 8**, and clinical trials in **Table 9**.

**Table 1:** Non-randomized clinical studies of proton versus photon radiation therapy – prostate cancer.

Study	Cancer Type	Interventions	Dose	Number of patients	Accrual Period	Follow up	Clinical Outcomes (Proton therapy/Carbon ion therapy vs Photon therapy)
(12) Sheets et al. (2012)	Nonmetastatic prostate cancer	PBT vs IMRT	not specified	684 PBT, 684 IMRT	2002-2007	PBT: M 50 mo (R 0.3 – 90.2 mo) IMRT: M 46 mo (R 0.4 – 88.3 mo)	GI morbidity: 17.8 vs 12.2 per 100 person-yrs* Urinary non-incontinence: 6.3 vs 7.5 per 100 person-yrs Urinary incontinence: 3.3 vs 3.1 per 100 person-yrs Erectile dysfunction: 7.4 vs 6.6 per 100 person-yrs Hip fracture: 0.7 vs 0.8 per 100 person-yrs Additional cancer therapy: 1.9 vs 2.2 per 100 person-yrs
(13) Yu et al. (2013)	Early-stage prostate cancer	PBT vs IMRT w/or w/o ADT	not specified	553 PBT, 27,094 IMRT	2008-2009	12 mo	GU toxicity: 5.9% vs 9.5% at 6 mo*, 18.8% vs 17.5% at 12 mo GI toxicity: 2.9% vs 3.6% at 6 mo, 9.9% vs 10.2% at 12 mo Other toxicity: <2.6% vs 2.5% at 6 mo, 4.5% vs 5.6% at 12 mo
(14) Pan et al. (2018)	Prostate cancer	PBT vs IMRT w/or w/o ADT	Dose not specified; PBT: M 39 fx IMRT: M 42 fx	693 PBT, 3465 IMRT	2008-2015	M 23 mo	Urinary toxicity: 33% vs 42% at 2y* Erectile dysfunction: 21% vs 28% at 2y* Bowel toxicity: 20% vs 15% at 2y*
(15) Hoppe et al. (2014)	Localized prostate cancer	PSPT vs IMRT w/or w/o ADT	PSPT: 78 - 82Gy at 1.8 – 2Gy/fx IMRT: 75.6 - 79.2Gy at 1.8-2Gy/fx	1,243 PSPT, 204 IMRT	PSPT: 2006-2010, IMRT: 2003-2006	24 mo	*Median EPIC scores:* Bowel domain: 0 vs 0 at 6 mo, -4 vs 0 at 1y, -4 vs 0 at 2y Urinary incontinence domain: 0 vs 0 at 6 mo, 0 vs 0 at 1y, 0 vs 0 at 2y Urinary irritative/obstructive domain: 0 vs 0 at 6 mo, 0 vs 0 at 1y, 0 vs 0 at 2y Sexual domain: 0 vs 0 at 6 mo, 0 vs 0 at 1y, 0 vs 0 at 2y
(16) Fang et al. (2015)	Localized prostate cancer	PSPT vs IMRT w/or w/o ADT	PSPT: 79.2Gy in 44 fx IMRT: not specified	181 PSPT, 213 IMRT	PSPT: 2010-2012, IMRT: 2009-2012	PBT: M 29 mo (R 5 – 50 mo) IMRT: M 47 mo (R 5 – 65 mo)	Grade ≥ 2 acute GI toxicity: 4.3% vs 13.8% Grade ≥ 2 late GI toxicity: 12.8% vs 10.8% Grade ≥ 2 acute GU toxicity: 21.3% vs 28.7% Grade ≥ 2 late GU toxicity:12.8% vs 18.3%
(17) Bai et al. (2020)	Stage T1-T2N0M0 prostate cancer	IMPT vs IMRT w/o ADT	60 Gy in 20 fx, 70.2 Gy in 26 fx, or 78 Gy in 39 fx	105 IMPT, 157 IMRT	2015-2018	not specified	*Mean EPIC Scores:* Bowel function domain: -6.7 vs -13 at end of treatment*, -1.2 vs -9.3 at 3 mo* Urinary incontinence domain: -2.6 vs -4.3 at the end of treatment, -0.4 vs -2.5 at 3 mo Urinary irritative/obstructive domain: -16.4 vs -16.2 at the end of treatment, 1.7 vs -2.4 at 3 mo
(18) Khmelevsky et al. (2018)	Stage T1-3N0-1M0 prostate cancer	Photon with PBT boost vs photon only w ADT	Photon: 44.0–46.0 Gy in 22–23 daily fx PBT boost: 28.0–28.8 Gy in 3.0 (8 daily fx), 4.0 (5 fx, 3/5 fx/wk), 5.5 (3 fx, 3 fx/wk) Gy Photon boost: up to 68.0–72.0 Gy in 12–14 fx at 2 Gy	116 PBT boost, 173 photon only	2000-2011	12-132 mo PBT boost: M 67.8 ± 3.1 mo Photon only: M 71.6 ± 2.9 mo	Acute GI toxicity: Grade 2: 54.4 ± 5.4% vs 69.2 ± 5.7%* Grade 3-4: 0 vs 0 Late GI toxicity: Grade 2: 10.2 ± 5.5% vs 34.8 ± 7.4% Grade 3-4: 0.9 ± 1.7% vs 1.3 ± 1.8% Acute GU toxicity: Grade 2: 33.3 ± 4.6% vs 36.1 ± 3.5%* Grade 3-4: 0% vs 1.9 ± 1.8% Late GU toxicity: Grade 2: 8.3% ± 5.0% vs 9.1 ± 4.5% Grade 3-4: 2.8% ± 2.6% vs 3.8 ± 3.0% 5y recurrence-free survival: 60.0 ± 5.4% vs 61.9 ± 4.4% 10y recurrence-free survival 45.5 ± 8.5% vs 42.8 ± 7.1% 5y OS: 74.0 ± 5.0% vs 78.8 ± 4.1% 10y OS: 55.9 ± 9.0% vs 60.6 ± 5.7%
(19) Liu et al. (2021)	Stage T1-3N0M0 prostate cancer	PBT vs 3D-CRT/IMRT	≥ 60 Gy PBT: mean (SD) 80.8 (24.7) 3D-CRT/IMRT: 79.2 (37.7)	620 PBT, 620 3D-CRT/IMRT	2004-2015	M 80.9 mo PBT: M 62.5 mo 3D-CRT/IMRT: M 76.5 mo	10y OS: 80.2% vs 71.3%*

Proton dose is RBE weighted.

*There is a statistically significant difference.

mo, month(s); wk, week(s); y, year(s); fx, fraction(s); M, median; R, range; w, with; w/o, without; vs, versus; PBT, proton beam therapy; PSPT, passively scattering proton therapy; IMRT, intensity modulated radiotherapy; IMPT, intensity modulated proton therapy; 3D-CRT, 3-D conformal radiation therapy; ADT, androgen deprivation therapy; GI, gastrointestinal; GU, genitourinary;

EPIC, expanded prostate cancer index composite; OS, overall survival.

### 3.1 Prostate cancer

Prostate cancer is the most common cancer diagnosed in men, with about 248,530 new cases and 34,130 deaths in the United States in 2021 (75). Since prostate cancer patients have a high long-term survival rate, minimizing treatment-related toxicities [gastrointestinal (GI) and/or genitourinary (GU)] and preserving quality of life (QoL) is a major goal of treatment.

A single institutional Medicare database propensity-matched study showed intensity-modulated radiation therapy (IMRT)-treated nonmetastatic prostate cancer patients had a lower incidence of GI toxicity than proton radiation therapy (12). The incidence of GU toxicity did not differ significantly between cohorts. However, Yu et al. (13) performed a multi-institutional study based on a national Medicare database and indicated there was a statistically significant reduction of GU morbidity rate at 6 months post-treatment in proton radiation therapy compared to IMRT and no difference at 12 months for early-stage prostate cancer patients after adjusting for potential confounders. Also, no statistically significant difference in GI morbidity was observed between groups. A claim-based propensity-matched study also indicated that proton radiation therapy was associated with lower incidences of urinary morbidity and erectile dysfunction, but a higher incidence of bowel morbidity at 2 years post-treatment, as compared to IMRT, among younger prostate cancer patients (< 65 years old) with private insurance (14). Since the Medicare database and medical claims could cause misclassification bias due to lack of detailed clinical information, Hoppe et al. (15) evaluated the patient-reported QoL between passive scattering proton therapy (PSPT) and IMRT for localized prostate cancer patients from nine University of Florida affiliated hospitals and found no differences in expanded prostate cancer index composite (EPIC)-26 summary scores for bowel, urinary, and sexual function domains between groups at 6 months to 2 years follow up after adjusting for potential confounders. A case-matched provider-reported-outcome study also showed no statistically significant differences between PSPT and IMRT for localized prostate cancer patients in acute or late grade ≥ 2 GI or GU toxicity rate within 5 years follow up, although planned doses to the bladder and rectum were significantly reduced in the PSPT group (16) (**Table 1**).

On the other hand, Bai et al. (17) assessed the patient-reported bowel and urinary toxicities between intensity-modulated proton therapy (IMPT) and IMRT at an early stage of post-treatment (immediately following and at 3 months post-treatment) for stage T1-2N0M0 prostate cancer patients in a single institution. Without adjusting for potential confounders, the IMPT group had a statistically smaller decline in EPIC-26 score for the bowel function domain than the IMRT group at both follow-up points and no difference for the urinary function domain. Without adjusting for confounders, Khmelevsky et al. (18) also presented that photon radiation therapy with a proton boost was associated with a statistically significantly lower incidence of acute and late grade 2 GI toxicity compared to photons only for patients with stage T1-3N0-1M0 prostate cancer. There were no statistically significant differences for acute and late grade 3-4 GI toxicity, acute and late GU toxicity, 5- and 10-year recurrence-free survival and OS between cohorts. Nevertheless, a propensity-matched study from the National Cancer Database reported proton-based treatment achieved higher 10-year OS than photon-based treatment (3D-conformal radiation therapy (3D-CRT)/IMRT) for stage T1-3N0M0 prostate cancer patients (19) (**Table 1**).

To date, there is only one randomized phase III study. This study, published in 1995, indicated that stage T3-4Nx,0-2M0 prostate cancer patients treated with high dose proton boost therapy experienced a significantly higher late treatment-induced rectal bleeding rate, but a lower local tumor persistence/palpable and/or symptomatic regrowth rate, as compared to the patients treated with conventional dose photon boost therapy (**Table 8**) (69). There were no statistically significant differences in acute grade 3-5 toxicity, late urinary toxicities, late sexual function, 8-year disease-specific survival, total recurrence-free survival and OS between groups. However, the patients with poorly differentiated tumors in the proton boost group experienced a significantly increased local control rate. Currently, three randomized clinical trials (NCT04190446: phase II, NCT01617161: phase III, NCT04083937: phase III) comparing treatment-related toxicities and QoL between IMRT and proton radiation therapy in prostate cancer are recruiting (**Table 9**).

### 3.2 Breast cancer

Breast cancer is the most commonly diagnosed cancer in women with an estimated 281,550 new cases and 43,600 deaths in the United States in 2021 (75). Radiation therapy complications include short-term (mainly skin toxicity) and long-term (such as ischemic heart disease, chronic radiation pneumonitis, nerve damage, etc.), and can negatively affect patient QoL.

Without adjusting for potential confounders, a multi-institutional prospective study indicated that PSPT resulted in a higher incidence of long-term (7-year) skin toxicities (telangiectasia, pigmentation change and other late skin toxicities) compared to 3D-CRT for stage I breast cancer patients (20). The 7-year local failure rate did not differ significantly between cohorts. A multivariable analysis based on the National Cancer Database revealed no statistically significant difference in 5-year OS between proton and photon therapies for stage 0-III breast cancer patients (21). On the other hand, a single institutional propensity-matched retrospective study showed pencil beam scanning proton therapy (PBSPT) treated patients had a higher incidence of acute grade ≥ 2 radiation dermatitis than photon radiation therapy treated patients with primary or recurrent stage IA-IIIC breast cancer, even though no statistically significant difference in skin dose was observed between groups (22). However, there were no statistically significant differences in acute grade ≥ 3 radiation dermatitis and acute grade ≥ 2 skin hyperpigmentation (**Table 2**).

**Table 2:** Non-randomized clinical studies of proton versus photon radiation therapy – breast cancer.

Study	Cancer Type	Interventions	Dose	Number of patients	Accrual Period	Follow up	Clinical Outcomes (Proton therapy vs Photon therapy)
(20) Galland-Girodet et al. (2014)	Stage I breast cancer	PSPT vs. 3D-CRT w/or w/o electron therapy	32 Gy in 8 fx twice daily	19 PSPT, 60 3D-CRT alone, 19 3D-CRT with electron therapy	2003-2006	M 82.5 mo (R 1.6-103.8 mo)	*Skin toxicity* at 7y: Telangiectasia: 69% vs 16%* Pigmentation changes: 54% vs 22%* Other late skin toxicities: 62% vs 18%* 7y Local failure: 11% vs 4%
(21) Chowdhary et al. (2019)	Stage 0-III breast cancer	PBT vs photon therapy w/or w/o electron boost w/or w/o chemotherapy	PBT: M 60.0Gy, photon therapy: M 60.4Gy	871 PBT, 723,621 photon therapy w/or w/o electron boost	2004-2014	M 62.2 mo	5y OS: 91.9% vs 88.9%
(22) DeCesaris et al. (2019)	Primary or recurrent stage IA-IIIC breast cancer	PBSPT vs photon therapy w/or w/o electron/proton/photon beam boost w/or w/o chemotherapy	PBSPT: R 45-50.4Gy with boost R 5.4-12.0Gy at 1.8 or 2.5Gy/fx, photon therapy: R 44-54Gy with boost R 9.0-26.0Gy at 1.8 or 2.5Gy/fx	39 PBSPT, 47 photon therapy	2015-2017	not specified	*Acute skin toxicity* Grade ≥ 2 RD: 69.2% vs 29.8%* Grade ≥ 3 RD: 5.1% vs 4.3% Grade ≥ 2 SH: 7.7% vs 12.8% Grade ≥ 3 SH: 0 vs 0

Proton dose is RBE weighted.

*There is a statistically significant difference.

mo, month(s); y, year(s); fx, fraction(s); M, median; R, range; w, with; w/o, without; vs, versus; 3D-CRT, 3-D conformal radiation therapy; PSPT, passively scattering proton therapy; PBT, proton beam therapy; PBSPT, pencil beam scanning proton therapy; OS, overall survival; RD, radiation dermatitis; SH, skin hyperpigmentation.

As discussed above, the published comparative clinical studies between proton and photon therapies for breast cancer are very limited and no comparative randomized clinical study has been published. Except skin toxicities, there were no published comparative studies investigating complications in other organs, such as lung and heart, where superior dose sparing in proton radiation therapy has been shown in numerous other studies. Three recruiting randomized clinical trials (NCT04443413: phase II, NCT04291378: phase III, NCT02603341) will primarily compare treatment-related heart disease and complication rates between proton and photon therapies in breast cancer (**Table 9**).

### 3.3 Lung cancer

Lung cancer, most commonly non-small cell lung cancer (NSCLC), continues to be the leading cause of cancer death for both men and women in the United States in 2021 (75). Thoracic radiation therapy can have unwanted side effects affecting nearby functional lung, heart, and esophagus, which can adversely affect QoL and survival. Therefore, the incidence of treatment-related pulmonary, cardiac, and esophageal complications must be considered when choosing the optimal treatment plan.

Two studies retrospectively analyzed the radiation-induced toxicities among patients enrolled in a randomized clinical trial (23, 24). Comparing PSPT and IMRT cohorts with stage II-IV NSCLC, these studies indicated the incidence of radiation-induced pericardial effusion and esophageal toxicity (based on either esophagitis grade distribution or esophageal expansion imaging biomarker values) did not differ significantly between cohorts. Remick et al. (25) also reported that no statistically significant differences in acute esophagitis incidence, 2-year OS, or local recurrence-free survival were achieved between double scattering proton therapy (DSPT)/IMPT and IMRT in stage I-IV NSCLC patients. However, without adjusting for potential confounders, Sejpal et al. (26) showed proton radiation therapy achieved lower incidence of acute grade ≥ 3 esophagitis and pneumonitis than 3D-CRT and IMRT for stage IB-IV and recurrent NSCLC patients, but no statistically significant difference for OS was found between cohorts. A recently published study also indicated PSPT/IMPT treated patients experienced a lower risk of acute grade ≥ 2 esophagitis and a trend of reducing the risk of acute grade ≥ 2 cardiac toxicity and acute grade ≥ 2 pneumonitis compared to IMRT treated patients with stage II-IV NSCLC using multivariate analysis (27). There were no statistically significant differences in 1-, 2-, and 5- year OS, progression-free survival, disease-specific survival, or local control between groups (**Table 3**).

**Table 3:** Non-randomized clinical studies of proton versus photon radiation therapy - lung cancer.

Study	Cancer Type	Interventions	Dose	Number of patients	Accrual Period	Follow up	Clinical Outcomes (Proton therapy vs Photon therapy)
(23) Cella et al. (2021)	Stage II-IV NSCLC	PSPT vs IMRT concurrent, induction or adjuvant chemotherapy	66 or 74 Gy	64 PSPT, 114 IMRT	2009-2014	M 24 mo (R 2-72 mo)	Pericardial effusion: 39% vs 46%
(24) Niedzielski et al. (2017)	Stage II-IV NSCLC	PSPT vs IMRT concurrent chemotherapy	60, 66 or 74 Gy in 2 Gy/fx over 6 – 8 wks	49 PSPT, 85 IMRT	not specified	not specified	Esophagitis: Grade 0: 18.4% vs 28.2%, Grade 2: 59.2% vs 54.1%, Grade 3: 22.4% vs 17.6%
(25) Remick et al. (2017)	Stage I-IV NSCLC	PBT (DSPT/PBSPT) vs IMRT neoadjuvant, sequential or concurrent chemotherapy	PBT: M 50.4 Gy (R 50.4-66.6 Gy) in 1.8 Gy/fx, once daily IMRT: M 54 Gy (R 50.0-72.0 Gy) in 1.8 Gy/fx, once daily	27 PBT (22 DSPT, 5 PBSPT), 34 IMRT	2011-2014	PBT: M 23.1 mo (R 2.3-42.0 mo) IMRT: M 27.9 mo (R 0.5-87.4 mo)	Tumor failure: 55.6% vs 61.8% 1y OS: 85.2% vs 82.4% 2y OS: 77.8% vs 73.2% 1y LRFS: 82.4% vs 93.3% 2y LRFS: 93.1% vs 85.7% *Acute toxicities:* Esophagitis: Grade 2: 18% vs 29% Grade 3: 4% vs 12% Pneumonitis: Grade 2: 4% vs 9% Grade 3: 4% vs 3%
(26) Sejpal et al. (2011)	Stage IB-IV + recurrent NSCLC	PBT vs 3D-CRT/IMRT concurrent chemotherapy	PBT: M 74 Gy, 3D-CRT, IMRT: M 63 Gy	62 PBT, 74 3D-CRT, 66 IMRT	PBT 2006-2008, 3D-CRT 2001-2003 or IMRT 2003-2005	PBT: M 15.2 mo (R 3.3-27.4 mo) 3D-CRT: M 17.9 mo (R 2.3-76.1 mo) IMRT: M 17.4 mo (R 1.8-65.5 mo)	*Acute toxicities:* Grade ≥ 3 esophagitis: 5% vs 18%/44%* Grade ≥ 3 pneumonitis: 2% vs 30%/9%* OS: M 24.4 mo vs 17.7 mo/17.6 mo
(27) Boyce-Fappiano (2021)	Stage II-IV NSCLC	PSPT/IMPT vs IMRT	M 54 Gy (R 45-74 Gy in 15-54 fx) PSPT/IMPT: M 54 Gy (R 45-74 Gy in 25-54 fx) IMRT: M 50 Gy (R 50-70 Gy in 25-37 fx)	61 PBT (55 PSPT, 6 IMPT), 75 IMRT	2003-2016	M 33.8 mo (R 1.3-179.2 mo) PSPT/IMPT: M 30.9 mo (R 1.3-136.1 mo) IMRT: M 40.6 mo (R 2.7-179.2 mo)	*Acute toxicities:* Grade ≥ 2 esophagitis 23% vs 60%* Grade ≥ 2 pneumonitis 4.9% vs 17% Grade ≥ 2 cardiac toxicity 4.9% vs 14.7% 1y-OS: 85.3% vs 89.3% 2y-OS: 66.5% vs 70.5% 5y-OS: 50.9% vs 37% 1y-PFS: 60% vs 67.2% 2y-PFS: 50.4% vs 46.7% 5y-PFS: 32.5% vs 36.9% 1y-DSS: 91.2% vs 97.2% 2y-DSS: 83.4% vs 89.6% 5y-DSS: 75.7% vs 60.2% 1y-LC: 89.1% vs 84.5% 2y-LC: 86.8% vs 82.7% 5y-LC: 83% vs 78.1%
(28) Zou et al. (2020)	Stage III lung cancer	USPT/PBSPT vs IMRT w/wo concurrent chemotherapy	>50 Gy, 1.8-2.0 Gy fx	34 PBT (6 USPT/28 PBSPT), 30 IMRT	2013 - 2018	M 16.8 mo (R 3.1- 63.8 mo USPT/PBSPT: M 16.1 mo IMRT: M 20.2 mo	Grade ≥ 2 esophagitis: 64.7% vs 53.3% Grade ≥ 2 pneumonitis: 20.6% vs 40% OS: M 41.6 mo vs 30.7 mo PFS: M 19.5 mo vs 14.6 mo LRC: 59.7% vs 44.2%
(29) Yu et al. (2020)	Stage I-IV NSCLC	IMPT vs IMRT w/wo concurrent chemotherapy	M 60 Gy (R 45-72 Gy) in M 30 (R 10-39) fx IMPT: M 2 Gy/d (R 1.9-5) IMRT: M 2 Gy/d (R 1.5-2)	33 IMPT, 46 IMRT	2016-2018	M 8.5 mo (R 1-27 mo)	*Subacute toxicities:* Grade 3 esophagitis: 6.1% vs 0 Grade 3 pneumonitis: 6.1% vs 2.2% Grade 3 dyspnea: 3.0% vs 6.5% 1y OS: 68 vs 65% 1y FFDM: 71 vs 68% 1y FFLR: 86 vs 69%
(30) Kim et al. (2019)	Stage I-II NSCLC	PBT (SBPT/IMPT) vs photon therapy (3D-CRT/SBRT/IMRT)	SBPT: 60–64 Gy in 4–8 fx IMPT: 60 Gy in 20 fx 3D-CRT and IMRT: 60 Gy in 20 fx over 4 wk or 15 fx over 3 wk	8 PBT (6 SBPT, 2 IMPT), 22 photon therapy (10 3D-CRT, 11 SBRT, 1 IMRT)	2010-2017	M 11 mo (R: 2-51 mo)	6 mo OS: 100% vs 67.9% 1y OS: 66.7% vs 46.4% Severe pulmonary toxicity: 12.5% vs 40.9%
(31) Kim et al. (2021)	Locally advanced NSCLC	PBSPT vs IMRT definitive concurrent chemoradiotherapy	66 Gy in 30 fx	29 PBSPT, 194 IMRT	2016-2018	M 23.0 mo (IQR 17.2–28.3 mo	SRL: 10.3% vs 35.6%*

Proton dose is RBE weighted.

*There is a statistically significant difference.

mo, month(s); wk, week(s); y, year(s); d, day(s); fx, fraction(s); M, median; R, range; IQR, Inter-quartile range; w, with; w/o, without; vs, versus; NSCLC, non-small cell lung cancer; 3D-CRT, 3-D conformal radiation therapy; IMRT, intensity modulated radiotherapy; SBRT, stereotactic body radiation therapy; PBT, proton beam therapy; PSPT, passively scattering proton therapy;

DSPT, double-scattering proton therapy; IMPT, intensity modulated proton therapy; USPT, Uniform scanning proton therapy; PBSPT, pencil beam scanning proton therapy; SBPT, stereotactic body proton therapy; OS, overall survival; LRFS, local recurrence-free survival; PFS, progression-free survival; LRC, locoregional control; FFDM, freedom from distant metastasis;

FFLR, freedom from locoregional recurrence; DSS, disease-specific survival; LC, local control; SRL, severe radiation-induced lymphopenia.

Comparing uniform scanning proton therapy (USPT)/PBSPT with IMRT, a single institutional study reported no statistically significant differences in grade ≥ 2 pneumonitis and esophagitis rates, acute dermatitis, OS, progression-free survival, or locoregional control for stage III lung cancer patients without adjusting for potential confounders (28). Yu et al. (29) also showed there were no statistically significant differences in the subacute (3 months post-treatment) grade 3 pneumonitis, esophagitis and dyspnea rates, 1-year OS, freedom from distant metastasis rate, or freedom from locoregional recurrence rate between IMPT and IMRT treated stage I-IV NSCLC patients using multivariable analysis. For early stage (I-II) NSCLC patients with underlying idiopathic pulmonary fibrosis (IPF), however, a multivariate analysis indicated there was a trend toward increased OS in the proton radiation therapy cohort (stereotactic body proton therapy (SBPT)/IMPT, 8 patients) compared to the photon cohort (stereotactic body radiation therapy (SBRT)/3D-CRT/IMRT, 22 patients) for 6-month and 1-year OS (30). Severe treatment-related pulmonary toxicity rates did not differ significantly between groups, possibly due to the small sample size of the study. However, 18.2% of patients in the photon radiation therapy group died of treatment-related pulmonary complications, but there were no treatment-related fatalities in the proton radiation therapy group. Another propensity-matched study from this institution reported that PBSPT treated patients had a lower risk of grade 4 radiation-induced lymphopenia than IMRT treated patients with locally advanced NSCLC (31) (**Table 3**).

A completed randomized phase II clinical trial (NCT00915005) confirmed that there was no statistically significant difference in radiation-induced pneumonitis between PSPT and IMRT for stage II-IV NSCLC patients (70, 71). In addition, another randomized phase II clinical trial (NCT01511081) showed that the SBPT group (10 patients) achieved better 3-year OS, progression-free survival and local control rate as compared to the SBRT group (9 patients) with early-stage (stage I or recurrent) NSCLC (72). However, this trial was terminated due to poor accrual numbers (**Table 8**). Three other randomized clinical trials (NCT02731001, NCT01993810: phase III, NCT01629498: phase I/II) comparing the toxicities and survival rates between photon and proton therapies in NSCLC are currently recruiting (**Table 9**).

### 3.4 Esophageal cancer

A single institutional retrospective study of stage I-IVA esophageal cancer treated using PSPT, 3D-CRT and IMRT showed a reduction in the incidence of postoperative pulmonary and GI complications between PSPT and 3D-CRT, but no statistically significant difference was found between PSPT and IMRT (32). A multi-institutional study also reported that PSPT treated patients had a lower rate of pulmonary and cardiac complications than 3D-CRT treated patients with stage I-IV esophageal cancer, whereas no statistically significant difference was found between PSPT and IMRT treated patients (33). Xi et al. (34) also showed that no significant grade ≥ 3 toxicity (mainly pulmonary, GI and cardiac complications) differences existed between PSPT/IMPT and IMRT for stage I-III esophageal cancer patients. However, the distant recurrence risk was significantly reduced, and the 5-year OS, progression-free survival and distant metastasis free survival were significantly improved in the proton group, especially in stage III esophageal cancer patients, using multivariate analysis. In contrast, without adjusting for potential confounders, Suh et al. (35) showed that no statistically significant differences in the 5-year progression-free survival, 5-year OS and the incidence of esophagitis, pneumonitis and pleural and pericardial effusion between PSPT/USPT/PBSPT and 3D-CRT/IMRT groups were seen in T1-3N0M0 thoracic esophageal cancer patients (**Table 4**).

**Table 4:** Non-randomized clinical studies of proton versus photon radiation therapy - esophageal cancer.

Study	Cancer Type	Interventions	Dose	Number of patients	Accrual Period	Follow up	Clinical Outcomes (Proton therapy vs Photon therapy)
(32) Wang et al. (2013)	Stage I-IVA esophageal cancer	PSPT vs 3D-CRT/IMRT w/or w/o chemotherapy	M 50.4 Gy at 1.8 Gy/fx	72 PSPT, 208 3D-CRT, 164 IMRT	PSPT: 2006-2011 3D-CRT: 1998–2008 IMRT: 2004–2011	not specified	Pulmonary complications: PSPT vs 3D-CRT: OR: 9.127, 95% CI:1.834-45.424* PSPT vs IMRT: OR: 2.228, 95% CI: 0.863-5.755 GI complications: PSPT vs 3D-CRT: OR: 2.311, 95% CI: 0.690-7.740* PSPT vs IMRT: OR: 1.025, 95% CI: 0.467-2.249
(33) Lin et al. (2017)	Stage I-IV esophageal cancer	PSPT vs 3D-CRT/IMRT w/wo induction chemotherapy	50.4 Gy at 1.8 Gy/fx	111 PSPT, 214 3D-CRT, 255 IMRT	2007-2013	not specified	Pulmonary complications: PSPT vs 3D-CRT: 16.2% vs 39.5%* PSPT vs IMRT: 16.2% vs 24.2% Cardiac complications: PSPT vs 3D-CRT: 11.7% vs 27.4%* PSPT vs IMRT: 11.7% vs 11.7%
(34) Xi et al. (2017)	Stage I-III esophageal cancer	PSPT/IMPT vs IMRT definitive chemotherapy	PSPT/IMPT: M 50.4 Gy (R: 45-63 Gy) in 28 fx IMRT: M 50.4 Gy (R 41.4-66 Gy) in 28 fx	132 PBT (125 PSPT, 7 IMPT), 211 IMRT	2007-2014	PSPT/IMPT: M 44.8 mo (R 11.9-110.3 mo) IMRT: M 65.1 mo (R 19.4-115.3 mo)	*Toxicities:* Pneumonitis: Grade 1: 7.6% vs 8.15% Grade 2: 2.3% vs 3.8% Grade 3: 0.8% vs 1.9% Grade 4: 0 vs 0.5% Grade 5: 0.8% vs 0.5% Esophagitis: Grade 1: 9.1% vs 11.8% Grade 2: 34.1% vs 31.3% Grade 3: 11.4% vs 14.2% Grade 4: 0 vs 0 Grade 5: 0vs 0.5% LRR: 33.3% vs 41.7% Distant recurrence: 33.3% vs 45% * 5y OS: 41.6% vs 31.6% * 5y PFS: 34.9% vs 20.4% * 5y DMFS: 64.9% vs 49.6% * 5y LRFFS: 59.9% vs 49.9%
(35) Suh et al. (2021)	T1–3N0M0 thoracic esophageal cancer	PBT (PSPT/USPT/PBSPT) vs 3D-CRT/IMRT w/wo concurrent chemotherapy	PBT: M 66 Gy (R 50–66 Gy), 3D-CRT/IMRT: M 64 Gy (R 56–66 Gy	48 PBT, 24 3D-CRT, 5 IMRT	2011-2019	PBT: M 25 mo (IQR 21–42 mo), 3D-CRT/IMRT: M 78 mo (IQR 69-97 mo)	5y OS: *P* = 0.52 5y PFS: *P* = 0.72
(36) Bhangoo et al. (2020)	Locally advanced esophageal cancer	IMPT vs IMRT concurrent chemotherapy	45Gy (R 41.4- 50.4Gy) in 25fx, with M 50Gy (R 50-56Gy) boost	32 IMPT, 32 IMRT	2014-2018	IMPT: M 10 mo, IMRT: M 14 mo	Acute grade 3 toxicity: 16% vs 9% 1y OS: 74% vs 71%
(37) DeCesaris et al. (2020)	Stage IIB-IVA distal esophageal cancer	PBSPT vs photon therapy concurrent chemotherapy	M 50.4Gy (R 41.4–50.4Gy) in 1.8Gy/fx	18 PBSPT, 36 photon therapy	2015–2018	PBSPT: M 18 mo, photon therapy: M 28 mo	18mo OS: 83% vs 59% 18mo LRC: 94% vs 92% 18mo DC: 79% vs 72%
(38) Shiraishi et al. (2018)	Stage I-IVA esophageal cancer	PBT vs IMRT w/wo induction chemotherapy	M 50.4Gy at 1.8Gy/fx	136 PBT, 136 IMRT	2005-2016	not specified	Grade 4 lymphopenia: 17.6% vs 40.4%*
(39) Routman et al. (2019)	Stage I-IV esophageal cancer	PBSPT vs 3D-CRT/IMRT concurrent chemotherapy	41.4-50.4 Gy	50 PBSPT, 50 3D-CRT/IMRT	2015-2017	not specified	Grade 4 lymphopenia: 24% vs 60%*

Proton dose is RBE weighted.

*There is a statistically significant difference.

mo, month(s); y, year(s); fx, fraction(s); M, median; R, range; w, with; w/o, without; vs, versus; 3D-CRT, 3-D conformal radiation therapy; IMRT, intensity modulated radiotherapy; PBT, proton beam therapy; PSPT, passively scattering proton therapy; IMPT, intensity modulated proton therapy; USPT, Uniform scanning proton therapy; PBSPT, pencil beam scanning proton therapy; OR, odds ratio; CI, confidence interval; GI, gastrointestinal; OS, overall survival; LRR, Locoregional recurrence; PFS, progression-free survival; LRFFS, locoregional failure-free survival; DMFS, distant metastasis-free survival; LRC, locoregional control; DC, distant metastatic control.

Comparing IMPT with IMRT, a single institutional study revealed that there were no statistically significant differences in acute grade 3 toxicity (mainly GI complication) and 1-year OS between groups for locally advanced esophageal cancer patients using multivariate analysis (36). Without adjusting for potential confounders, DeCesaris et al. (37) also reported that 18-month OS, locoregional control and distant metastatic control were similar between PBSPT and photon treated patients with stage IIB-IVA distal esophageal cancer. However, there were two propensity-matched studies showed that proton radiation therapy (PBSPT) was associated with a significantly lower incidence of grade 4 lymphopenia compared to 3D-CRT/IMRT for stage I-IV esophageal cancer patients (38, 39) (**Table 4**).

The only published randomized phase IIB clinical study (NCT01512589) reported that the significant dose sparing of lung, heart, liver and lymphocytes in a PSPT/IMPT group resulted in reduced total toxicity burden and postoperative complications scores compared to an IMRT group for stage I-III esophageal cancer patient, but the QoL, 3-year progression-free survival and OS were similar between groups (**Table 8**) (73). Two additional ongoing randomized phase III clinical trials (NCT05055648, NCT03801876) will attempt to clarify the superior safety and efficacy of proton radiation therapy for esophageal cancer in the next several years (**Table 9**).

### 3.5 Head and neck cancers

For stage T1-4N0-3 nasopharynx cancer, a retrospective case-matched study showed that IMPT treated patients had a lower requirement for gastrostomy tube placement than IMRT treated patients, which is likely driven by the lower dose to the oral cavity from IMPT (40). Similarly, a multivariate analysis from McDonald et al. (41) reported that proton radiation therapy for stage T1-4N0-2 nasopharynx and paranasal sinus cancer patients resulted in a lower requirement for gastrostomy tube insertion and opioid pain medication at the end of radiation therapy and one month post-treatment, which may also be due to the significant mean dose reduction to oral cavity, esophagus, larynx, and parotid glands, as compared to IMRT. To compare the radiation-related toxicities and survival rates between IMRT only and IMRT with PBSPT boost, Alterio et al. (42) analyzed outcomes of stage T3-4N0-2 nasopharyngeal carcinoma patients and showed that patients treated with a PBSPT boost experienced significantly lower risk of acute grade 3 mucositis and acute grade 2 xerostomia. However, no statistical differences were found for late toxicities, local progression-free survival, progression-free survival and local control rate between groups (**Table 5**).

**Table 5:** Non-randomized clinical studies of proton versus photon radiation therapy – head and neck cancer.

Study	Cancer Type	Interventions	Dose	Number of patients	Accrual Period	Follow up	Clinical Outcomes (Proton therapy vs Photon therapy)
(40) Holliday et al. (2015)	Stage T1-4N0-3 nasopharyngeal cancer	IMPT vs IMRT w/or w/o induction chemotherapy	70 Gy in 33-35 fx of 2-2.12 Gy/fx	10 IMPT, 20 IMRT	2011-2013	IMPT: M 21.6 mo (IQR 13.6-28.6 mo) IMRT: M 25.8 mo (IQR 17.2-36.7 mo)	GT insertion: 20% vs 65% * CTC grade 3 acute toxicities: 50% vs 90%* Dermatitis: Grade 1 dermatitis: 10% vs 35% Grade 2 dermatitis: 40% vs 40% Grade 3 dermatitis: 40% vs 25 Weight loss: 5.7% vs 7.6% Swallowing dysfunction: 0 vs 15%
(41) McDonald et al. (2016)	Stage T1-4N0-2 nasopharynx, nasal cavity or paranasal sinuses cancer	PBT vs IMRT w/or w/o chemotherapy	PBT: M 71.4 Gy (R 63-75.6 Gy) IMRT: M 71.8 Gy (R 66-76.4 Gy)	14 PBT, 26 IMRT	2010-2014	not specified	GT dependent: *P* < 0.001 at the end of RT*; *P* = 0.033 at 1 mo* EMD > baseline: *P* = 0.006 at the end of RT*
(42) Alterio et al. (2020)	Stage T3–4N0–2 nasopharyngeal cancer	IMRT with PBSPT boost vs IMRT only Induction chemotherapy	IMRT up to 54-60 Gy with PBSPT up to 70-74 Gy IMRT only: M 70 Gy (R 68-70 Gy)	27 PBSPT boost, 17 IMRT only	PBSPT boost: 2012-2017 IMRT only: 2006-2015	not specified	*Acute toxicities:* Skin: *P* = 0.66 Mucositis: *P* = 0.0002* Dysphagia: *P* = 0.36 Xerostomia: *P* = 0.02* Weight loss: *P =* 0.11 Enteral nutrition: *P* = 0.81 Dysphonia: *P =* 0.06 Hearing impairment: *P* = 0.64 Dysgeusia: *P* = 0.55, Pain: *P* = 0.34 *Late toxicities:* Skin: *P* = 0.55, Mucositis: *P* = 0.20 Dysphagia: *P* = 1 Xerostomia: *P* = 0.15 Cranial nerve neuropathy: *P =* 0.12 Trismus: *P =* 0.51 Hearing impairment: *P* = 0.38 Dysgeusia: *P* = 0.71 CNS necrosis: *P =* 1 Soft tissue necrosis: *P =* 0.38 Soft tissue fibrosis: *P =* 0.07 Optic nerve disorder: *P =* 0.77 Endocrine disorders: *P =* 0.61 LPFS: *P =* 0.17 PFS: *P =* 0.4 Local control: 96% IMRT with IMPT boost vs 81% IMRT only
(43) Sio et al. (2016)	Stage T1-4N0-3 oropharyngeal cancer	IMPT vs IMRT concurrent chemotherapy	IMPT: M 70 Gy (R 59-70 Gy) IMRT: M 70 Gy (R 58-70 Gy)	35 IMPT, 46 IMRT	2006-2015	IMPT: M 7.7 mo (IQR 3.97–22.77 mo) IMRT M 2.68 mo (IQR 0.30–10.27 mo)	MDASI-NH mean top 5 symptom scores: 5.15 ± 2.66 vs 6.58 ± 1.98, *P*= 0.013*
(44) Blanchard et al. (2016)	Stage T1-4N0-3 oropharynx cancer	IMPT vs IMRT w/wo induction chemotherapy	66 Gy or 70 Gy or 54–63 Gy.	50 IMPT, 100 IMRT	2010–2014	M 32 mo (R 2–55 mo) IMPT: M 29 mo (R 8–49 mo) IMRT: M 33 mo (R 2–55 mo)	*3mo post-RT:* Grade 3 weight loss or GT: 18% vs 34%* Grade ≥ 2 xerostomia: 42% vs 61.2%* Grade ≥ 2 fatigue: 40.8% vs 36.2% *1y post-RT:* Grade 3 weight loss or GT: 8% vs 24.7%* Grade ≥ 2 xerostomia: 42% vs 47.2% Grade ≥ 2 fatigue: 14.6% vs 22.1% 3y OS: 94.3% vs 89.3% 3y PFS: 86.4% vs 85.8% 3y LRC: 91.0% vs 89.7% 3y DC: 97.8% vs 93.5%
(45) Sharma et al. (2018)	Stage I-IVA oropharynx cancer	PBSPT vs IMRT/VMAT w/wo chemotherapy	60 to 66 Gy	31 PBSPT, 33 IMRT/VMAT	2013-2015	up to 12 mo	*3mo post-RT:* Xerostomia: 50% vs 47.62% H&N pain: 25% vs 28.85% Fatigue: 26.5% vs 26.5% *6mo post-RT:* Xerostomia: 39.58% vs 52.63% H&N pain: 8.33% vs 18.86% Fatigue: 8.5% vs 20.47% *1y post-RT:* Xerostomia: 23.53% vs 54.55%* H&N pain: 8.33% vs 21.97%* Fatigue: 4.86% vs 22.22%
(46) Romesser et al. (2016)	Major salivary gland cancer or cutaneous squamous cell carcinoma	USPT vs IMRT	USPT: M 66Gy (IQR 61.2-66 Gy) IMRT: M 66 Gy (IQR 66-66Gy)	18 USPT, 23 IMRT	2011-2014	USPT: M 4.7 mo (IQR 1.6–7.9 mo) IMRT: M 16.1 mo (IQR 8.7–24.4 mo)	*Grade ≥ 2 acute toxicities:* Dysgeusia: 5.6% vs. 65.2%*, Mucositis: 16.7% vs. 52.2%*, Nausea: 11.1% vs. 56.5%* 1y actuarial DMFS: 83.3% vs 93.3% 1y actuarial OS: 83.3% vs 93.3% 1y actuarial LRC: 80% vs 95.5%

Proton dose is RBE weighted.

*There is a statistically significant difference.

mo, month(s); y, year(s); fx, fraction(s); M, median; R, range; IQR, interquartile range; w, with; w/o, without; vs, versus; IMRT, intensity modulated radiotherapy; IMPT, intensity modulated proton therapy; USPT, uniform scanning proton therapy; PBT, proton beam therapy; PBSPT, pencil beam scanning proton therapy; VMAT, volumetric modulated arc therapy;

MDASI-HN, MD Anderson symptom inventory-head and neck module; LRC, locoregional control; PFS, progression-free survival; DC, distant control; GT, gastrostomy tube; RT, radiation therapy; EMD, equivalent morphine dose; CTC, common terminology criteria; CNS: central nervous system; H&N, head and neck; LRFS, local recurrence-free survival; PFS, progression-free survival; DMSF, distant metastasis-free survival; OS, overall survival.

For stage T1-4N0-3 oropharyngeal cancer, a retrospective patient-reported outcome study from a symptom inventory-head and neck (MDASI-HN) module survey at MD Anderson indicated that there was a statistical reduction of top 5 symptom scores (food taste, dry mouth, swallowing/chewing, fatigue and appetite) in the IMPT group compared to the IMRT group during the subacute phase (first 3 months post- treatment) without adjusting for potential confounders (43). However, the top 11 symptom scores (food taste, dry mouth, swallowing/chewing, fatigue, appetite, mucus, sleep, mouth sores, drowsiness and distress) did not differ significantly between groups during the acute phase (6- to 7-week period during treatment) and chronic phase (after 3 months post-treatment). Meanwhile, a case-matched study reported that IMPT was associated with a lower incidence of patient-reported grade ≥ 2 xerostomia at 3 months post-treatment and a lower risk of grade 3 weight loss or gastrostomy tube presence at one year post-treatment compared to IMRT for stage T1-4N0-3 oropharyngeal cancer patients (44). However, there were no significant differences in patient-reported grade ≥ 2 dermatitis or mucositis and fatigue, 3-year OS, progression-free survival, locoregional control rate and distant control rate between groups. Another patient-reported outcome study also indicated that PBSPT treated patients had a statistically significant lower xerostomia score and less head and neck pain than IMRT/volumetric modulated arc therapy (VMAT) treated patients with stage I-IVA oropharynx cancer at one year post-treatment, which is likely due to significant dose sparing of the oral cavity structure (45) (**Table 5**).

To compare radiation-induced toxicities between uniform scanning proton therapy (USPT) and IMRT for major salivary gland cancer or cutaneous squamous cell carcinoma, Romesser et al. (46) showed USPT was associated with a lower risk of acute grade ≥ 2 treatment-related toxicities (mucositis, dysgeusia, and nausea) compared to IMRT for patients who received ipsilateral head and neck radiation, which may be a result of the significant dose sparing of oral cavity and brainstem. However, 1-year actuarial locoregional control rate, actuarial distant metastasis-free survival, and actuarial OS did not differ significantly between cohorts (**Table 5**). To date, no comparative randomized clinical study for head and neck cancers has been published, but many randomized clinical trials (3 phase II, 1 phase II/III, 2 not specify) comparing treatment-induced toxicities between photon and proton therapies are currently recruiting (**Table 9**).

### 3.6 Central nervous system cancer

#### 3.6.1 Adult CNS cancer

For craniospinal irradiation (CSI), Gunther et al. (47) reported that PSPT CSI treated patients experienced a lower risk of acute grade 1-3 mucositis than 3D-CRT-CSI treated patients with leukemia/lymphoma/myeloma with CNS involvement/elapse. No statistically significant differences in acute GI symptoms, CNS toxicity/relapse, infection, or 6-month survival rate were observed between cohorts. However, Brown et al. (48) demonstrated proton CSI was associated with a lower risk of acute GI and hematologic morbidities compared to photon CSI for stage M0-4 medulloblastoma patients. The reduced risk of acute GI morbidities in proton CSI patients, including weight, grade 2 nausea/vomiting, and esophagitis-related medical management, was most likely due to the significant dose sparing of the esophagus, stomach, and bowel. The reduced risk of acute hematologic morbidities (bone marrow suppression) in proton CSI patients, including less decline of peripheral white blood cells (WBC), hemoglobin, and platelets, was mainly driven by the significantly lower mean vertebral dose. Proton CSI patients also had a significantly lower incidence of grade ≥ 1 anemia than photon CSI patients, whereas the incidence of grade ≥ 1 leukopenia and thrombocytopenia did not differ significantly (**Table 6**).

**Table 6:** Non-randomized clinical studies of proton versus photon radiation therapy – adult CNS cancer.

Study	Cancer Type	Interventions	Dose	Number of patients	Accrual Period	Follow up	Clinical Outcomes (Proton therapy vs Photon therapy)
(47) Gunther et al. (2017)	Leukemia/lymphoma/myeloma patients with CNS involvement/relapse	PSPT vs 3D-CRT	PSPT: M 21.8 Gy (IQR 21.3-23.6 Gy),3D-CRT: M 24 Gy (IQR 23.4–24)	14 PSPT, 23 3D-CRT	2011-2015	M 8 mo (IQR 6–17.5 mo)	*During CSI:* Grade 1-3 mucositis: 7% vs 44%* Infection: 57% vs 35% GI toxicity: 29% vs 30% CNS toxicity: 21% vs 13% *During SCT:* Mucositis: 50% vs 48% Infection: 86% vs 87% Neutropenic fever: 29% vs 57% GI toxicity: 79% vs 70% CNS toxicity: 29% vs 35% CV toxicity: 29% vs 30% Pulmonary toxicity: 21% vs 17% 6mo OS: 69.6% vs 78.6%
(48) Brown et al. (2013)	Stage M0-4 medulloblastoma	PBT vs photon therapy	54 Gy	19 PBT, 21 photon therapy	2003-2011	PBT: M 26 mo (R 11-63 mo) Photon therapy: M 57 mo (R 4-103 mo)	*Acute GI toxicities:* Weight lose: 1.2% vs 5.8%* ≥ 5% weight lose: 16% vs 64%* Grade 2 nausea/vomiting: 26% vs 71%* Esophagitis medical management: 5% vs 57%* Intravenous fluid support: 0 vs 14% *Acute hematologic toxicities*: WBC reduction: 46% vs 55%* Hb reduction: 88% vs 97%* Platelet reduction: 48% vs 65%* Grade ≥ 1 anemia: 17% vs 48%* Grade ≥ 1 leukopenia: 84% vs 77% Grade ≥ 1 thrombocytopenia: 12% vs 29% 2y OS: 94% vs 90% PFS: 94% vs 85%
(49) Song et al. (2021)	Grade I-III meningioma	USPT/PBSPT vs VMAT/Tomotherapy	M 54 Gy (R 50-60 Gy) at M 1.8 Gy/fx (R 1.8-2.3 Gy/fx) in M 30 fx (R 25-33 fx), USPT/PBSPT: M 54 Gy (R 50.4-60 Gy) at M 1.8 Gy/fx (R 1.8-2 Gy/fx) in M 30 fx (R 28-33 fx), VMAT/Tomotherapy: M 54 Gy (R 50-60 Gy) at M 2 Gy/fx (R 1.8-2.3 Gy/fx) in M 27 fx (R 25-33 fx)	15 USPT/23PBSPT, 32 VMAT/7 Tomotherapy	USPT/PBSPT 2014-2017, VMAT/Tomotherapy 2008-2018	M 2.2y, USPT/PBSPT: M 1.7y, VMAT/Tomotherapy: M 3.1y	Grade ≥ 2 symptomatic brain injury: 7.7% vs 10.5% 2y PFS: 76% vs 81.3% 2y OS: 86.6% vs 89.3%
(50) Jhaveri et al. (2018)	Grade I-IV glioma	PBT vs photon therapy	M 60 Gy	170 proton therapy, 49,405 PBT	2004-2013	M 62.1 mo, PBT: M 50.3 mo, photon therapy: M 62.3 mo	5y OS: 46.1% vs 35.5%*

Proton dose is RBE weighted.

*There is a statistically significant difference.

mo, month(s); y, year(s); fx, fraction(s); M, median; R, range; IQR, interquartile range; w, with; w/o, without; vs, versus; PBT, proton beam therapy; 3D-CRT, 3-D conformal radiation therapy; PSPT, passively scattering proton therapy; USPT, Uniform scanning proton therapy; PBSPT, pencil beam scanning proton therapy; VMAT, volumetric modulated arc therapy; CSI, craniospinal irradiation; CNS, central nervous system; GI, gastrointestinal; SCT, stem cell transplantation; CV, cardiovascular; OS, overall survival; PFS, progression-free survival; WBC, white blood cells; Hb, hemoglobin.

For cranial irradiation, Song et al. (49) showed there were no statistically significant differences in grade ≥ 2 symptomatic brain injury, 2-year progression-free survival and OS between USPT/PBSPT and VMAT/tomotherapy for grade I-III meningioma. However, a retrospective study based on the National Cancer Database indicated that grade I-IV glioma patients treated with proton radiation therapy achieved superior 5-year OS compared to photon radiation therapy after propensity score weighting (50) (**Table 6**). Recently, a completed randomized phase II clinical trial (NCT01854554) for glioblastoma reported that patients treated with PSPT/IMPT had a statistically reduced rate of acute grade ≥ 3 lymphopenia compared to IMRT/VMAT, which is likely due to the reduced brain volume irradiated by low and intermediate doses (**Table 8**) (74).

As listed above, the comparative clinical studies between proton and photon therapies for adult CNS cancer are limited. Ongoing randomized clinical trials (NCT04752280, NCT03180502: phase II, NCT02179086: phase II, NCT04536649: phase III) may provide additional clinical evidence to clarify the effectiveness of proton radiation therapy (**Table 9**).

#### 3.6.2 Pediatric CNS cancer

Due to the high radiosensitivity of developing tissues and the long life-expectancy of childhood cancer survivors, severe long-term side effects and radiation-induced secondary malignancies are major concerns when treating pediatric cancer patients with radiation. Therefore, sufficient avoidance of non-target tissues to mitigate treatment-related toxicities is crucial for treatment planning.

For cognitive development following CSI, Kahalley et al. (51) compared intelligence quotient (IQ) change over time between PSPT/IMPT and 3D-CRT/IMRT following treatment of pediatric brain cancer patients in a single institutional study. The study reported that there was no significant IQ decline over time from proton radiation therapy, while photon radiation therapy patients exhibited a significantly lower and steadily decreasing IQ score for both craniospinal and focal irradiation. Subgroup evaluation also indicated that photon CSI is associated with a reduced IQ of 12.5 points compared to proton CSI. The authors did not mention the IQ comparison between focal photon and proton therapies. The authors further evaluated different domains of intellectual outcomes from a multi-institution database and determined that patients treated with proton CSI had better intellectual outcomes in global IQ, perceptual reasoning, and working memory as compared to photon CSI (52). The verbal reasoning score did not differ between cohorts and patients in both cohorts experienced a significant reduction in processing speed score. However, Eaton et al. (53) recently indicated that PSPT CSI-treated patients had higher verbal reasoning score, mean full-scale IQ, and perceptual reasoning score as compared to 3D-CRT/IMRT CSI-treated patients with standard-risk pediatric medulloblastoma in a multi-institutional case-matched study. No statistically significant differences in processing speed and working memory were detected between groups. Gross et al. (54) also compared different intellectual parameters and reported pediatric brain cancer patients treated with PSPT/IMPT showed higher full-scale IQ and processing speed index than those treated with 3D-CRT/IMRT for both craniospinal and focal irradiation. The subgroup investigation indicated that pediatric patients treated with proton CSI achieved higher full-scale IQ and verbal IQ than those treated with photon CSI, and proton focal irradiation resulted in higher processing speed index than photon focal irradiation. However, a recently published long-term (average 7.2 years post-treatment) study compared cognitive and academic outcomes between craniospinal and focal proton and photon therapies for pediatric primary brain cancer patients and showed there were no statistically significant differences in full-scale IQ, verbal comprehension, perceptual reasoning, working memory and processing speed index between cohorts (55) (**Table 7**).

**Table 7:** Non-randomized clinical studies of proton versus photon radiation therapy – pediatric CNS cancer.

Study	Cancer Type	Interventions	Dose			Number of patients	Accrual Period	Follow up	Clinical Outcomes (Proton therapy vs Photon therapy)
(51) Kahalley et al. (2016)	Pediatric brain tumor	PSPT/IMPT vs 3D-CRT/IMRT	PSPT/IMPT: M 54 Gy (R 30-60 Gy) 3D-CRT/IMRT: M 54 Gy (R 30.6-59.4 Gy)			90 PBT (81 PSPT, 9 IMPT), 60 3D-CRT/IMRT	PSPT/IMPT: 2007-2012 3D-CRT/IMRT 2002-2007	not specified	IQ: 3D-CRT/IMRT vs PSPT/IMPT (- 8.7 points average, *P* = 0.011)*
(52) Kahalley et al. (2020)	pediatric medulloblastoma	PBT vs photon therapy with chemotherapy	standard-dose 30.6-39.6 Gy or reduced dose 15.0-23.4 Gy to the whole brain and spine			37 PBT, 42 photon therapy	2007-2018	not specified	Global IQ: *P* = 0.011* PIQ: *P* = 0.022* Working memory: *P* = 0.002* VIQ: *P* > 0.05 PSI: *P* > 0.05
(53) Eaton et al. (2021)	standard-risk Pediatric medulloblastoma	PSPT vs 3D-CRT/IMRT	CSI dose: PSPT: M 23.4Gy (R 18-27Gy), 3D-CRT/IMRT: M 23.4Gy (R 18-26.4Gy)			25 PSPT, 25 3D-CRT/IMRT	2000-2009	PSPT: M 5.3y (R 1.0-11.4y), 3D-CRT/IMRT: M 4.6y (R 1.1-11.2y)	FSIQ: 99.6 vs 86.2* VIQ: 105.2 vs 88.6* PIQ: 103.1 vs 88.9* PSI: 82.9 vs 77.2 Working memory: 97.0 vs 92.7
(54) Gross et al. (2019)	pediatric brain tumor	PSPT/IMPT vs 3D-CRT/IMRT	not specified			58 PBT (11 PSPT, 47 IMPT), 67 photon therapy (26 3D-CRT, 41 IMRT)	1998-2017	M 3.2y (IQR 1.8-4.7y)	FSIQ/GAI: *P* = 0.048* PSI: *P* = 0.007* VIQ: *P* = 0.06 *Adaptive functioning across domains:* GAC *P* = 0.07 Conceptual: *P* = 0.09 Social: *P* = 0.07 Practical: *P* = 0.08 *Focal irradiation:* PSI: *P* = 0.01* *CSI:* FSIQ/GAI: *P* = 0.01* VIQ: *P* = 0.01*
(55) Child et al. (2021)	pediatric primary brain tumor	PBT vs photon therapy	Focal PBT: M 50.4Gy (R 45.0–59.4Gy), Focal photon therapy: M 54.0Gy (R 48.6–59.4Gy), CSI PBT: M 54.0Gy (R 45.0–55.8Gy), CSI photon therapy: M 54.0Gy (R 30.6–55.8Gy)			58 PBT, 30 photon therapy	PBT: 2007-2013, photon therapy: 2001-2006	Focal PBT: M 6.3 ± 2.7y (R 1.2–10.6y), Focal photon therapy: M 8.7 ± 3.4y (R 4.0–15.3y), CSI PBT: M 5.9 ± 3.3y (R 1.2–11.1y), CSI photon therapy: M 9.8 ± 2.5y (R 5.8–13.9y)	*Focal irradiation:* FSIQ: 99.0 ± 2.7 vs 92.5 ± 4.3 VIQ: 101.8 ± 2.2 vs 101.4 ± 3.6 PIQ: 103.6 ± 3.2 vs 96.3 ± 5.1 Working memory: 96.4 ± 2.9 vs 95.8 ± 4.5 PSI: 87.9 ± 3.2 vs 78.5 ± 5.1 *CSI:* FSIQ: 86.3 ± 4.5 vs 71.3 ± 7.3 VIQ: 90.2 ± 4.4 vs 80.0 ± 7.1 PIQ: 92.7 ± 5.2 vs 76.9 ± 8.4 Working memory: 89.0 ± 4.4 vs 77.8 ± 7.1 PSI: 76.0 ± 2.8 vs 72.3 ± 4.4
(56) Bielamowicz et al. (2018)	Standard and high risk pediatric medulloblastoma	PSPT vs 3D-CRT+IMRT boost w/chemotherapy	PSPT: M 55.8Gy (R 36-57Gy),3D-CRT+IMRT boost: M 55.8Gy (R 54-59.4Gy)			41 PSPT, 54 3D-CRT+IMRT boost	1997-2014	PSPT: M 3.8y (R 1.0-8.8y),3D-CRT+IMRT boost: M 9.6y (R 1.0-15.8y)	Primary hypothyroidism: 7.3 vs 20.4% Central hypothyroidism: 9.8 vs 24.0%
(57) Aldrich et al. (2021)	Pediatric medulloblastoma	PSPT vs 3D-CRT+IMRT boost	standard/low-risk: 15-23.4Gy, high risk: 36-39.6Gy			64 PSPT, 54 3D-CRT+IMRT boost	1997-2016	M 5.6y (R 1.0-10.0y)	Primary hypothyroidism: 6% vs 28%*
(58) Eaton et al. (2016)	Standard risk pediatric medulloblastoma	PSPT vs 3D-CRT/IMRT w/chemotherapy	54–55.8 Gy at 1.8Gy/fx, or 60Gy in 1.2Gy/fx			40 PSPT, 37 photon therapy (13 3D-CRT, 24 IMRT)	2000-2009	PSPT: M 5.8y (R 3.4–9.9y), 3D-CRT/IMRT: M 7.0y (R 3.5–13.5y)	Hypothyroidism: 23% vs 69%* Sex hormone deficiency: 3% vs 19%* Endocrine replacement therapy requirement: 55% vs 78%* Height standard deviation score: 21.19 ( ± 1.22) vs 22 ( ± 1.35)* Growth hormone deficiency: 53% vs 57% Adrenal insufficiency: 5% vs 8% Precocious puberty: 18% vs 16%
(59) Liu et al. (2021)	Pediatric medulloblastoma	DSPT vs photon therapy w/or w/o concurrent chemotherapy	DSPT: M 54 Gy (R 54-55.8 Gy) Photon therapy: M 54 Gy (R 52.2-55.8 Gy)			60 DSPT, 37 photon therapy	2000-2017	DSPT: M 8.1y (R 0.2-13.7y); Photon therapy: M 7.1y (R 0.2-17.5y)	*Hematologic toxicity:* Leukopenia: *P* = 0.044* Neutropenia: *P* = 0.762 Lymphopenia: *P* < 0.0001* Anemia: *P* = 0.011* Thrombocytopenia: *P* = 0.066 5y OS: 89.6% vs 93.4%
(60) Song et al. (2014)	Pediatric brain tumor (mainly medulloblastoma)	PBT vs Photon therapy	PBT: mean 29.4 Gy (R 19.8-39.6 Gy) at 1.8 Gy/fx Photon therapy: mean 32.1 Gy (R 23.4-39.6 Gy) at 1.8 or 1.5 Gy/fx			30 PBT, 13photon therapy	PBT: 2008-2012 Photon therapy: 2003-2012	M: 22 mo (R 2-118 mo)	*GI toxicity:* Nausea: 33% vs 46% Dysphagia: 47% vs 15% Anorexia: 37% vs 31% Vomiting: 30% vs31% Diarrhoea: 0 vs 23%* *Hematologic toxicity:* Leukopenia: 64% vs 78% Anaemia: 0 vs 15% Thrombocytopenia: 23% vs 54%* Platelet transfusion: 17% vs 46%* RBC transfusion: 50% vs 39% WBC: -0.57 ± 2.22 vs -2.61 ± 2.27* Hb: +0.23 ± 1.04 vs -0.7 ± 1.89 Platelet: -0.49 ± 0.64 vs -1.37 ± 0.96*
(61) Yoo et al. (2022)	Pediatric brain tumors	PBSPT vs 3D-CRT/helical tomotherapy	up to 30.6Gy at 1.5 or 1.8Gy/fx in M 13fx (R 10-17fx)			36 PBSPT, 29 3D-CRT/1 helical tomotherapy	2010-2019	M 38 mo (R 1-114 mo)	Hb: *P* = 0.328 ALC: *P* = 0.018* PLT: *P* = 0.007* Diarrhea: 0 vs 3.3% Grade 3 anemia: 0 vs 13.3%* Grade 4 lymphopenia: 30.6% vs 43.3% Grade 3 thrombocytopenia: 11.1% vs 20% Platelet transfusion: 5.6% vs 13.3% 3y OS: 92.9% vs 93.2%
(62) Paulino et al. (2021)	Pediatric medulloblastoma	PSPT vs 3D-CRT+IMRT boost w/chemotherapy	CSI dose: 18.0-23.4Gy or 30.6-40Gy			52 PSPT, 63 IMRT	1996-2014	PSPT: M 8.7y (R 0.4-13.4y), IMRT: M 12.8y (R 0.2-20.3y)	5y OS: 80.3% vs 80% 10y OS: 72.4% vs 78.1% 5y SMN: 2.2% vs 0 10y SMN: 4.9% vs 8%
(63) Eaton et al. (2016)	Pediatric standard risk medulloblastoma	PBT vs 3D-CRT/IMRT w/chemotherapy	M 23.4 Gy (R 18-27 Gy), boost 30.6 Gy (R 27-37.8 Gy)			45 PBT, 43 3D-CRT/IMRT	2000-2009	PBT: M 6.2 y (R 5.1-6.6 y) 3D-CRT/IMRT: M 7 y (R 5.8-8.9 y)	6y OS: 82% vs 87.6% 6y RFS: 78.8% vs 76.5% Patterns of failure: 22.2% vs 23.3%
(64) Paulino et al. (2018)	Pediatric medulloblastoma	PSPT vs 3D-CRT+IMRT boost w/chemotherapy	18–23.4 Gy for standard-risk patients, 36–39.6 Gy for high-risk patients			38 PSPT, 46 3D-CRT+IMRT boost	1997-2013	PSPT: M 56mo (R 13–101 mo), 3D-CRT+IMRT boost: M 66mo (R 13–163 mo)	Grade 3&4 hearing loss SIOP Boston scale: 20% vs 23.1% Brock scale: 9.3% vs 9% POG scale: 17.3% vs 20.9% CTCAE scale: 29.9% vs 28.3%
(65) Trybula et al. (2021)	Pediatric medulloblastoma	PBT vs Photon therapy	PBT: 54.8Gy, Photon therapy: 54.2Gy			49 PBT, 30 Photon therapy	2003-2019	PBT: 56.8 mo, Photon therapy: 105 mo	CM: 85.7% vs 86.7%
(66) Bishop et al. (2014)	Pediatric craniopharyngioma	PBT (mainly PSPT) vs IMRT	PBT and IMRT: 50.4-54 Gy at 1.8 Gy/fx			21 PBT (18 PSPT), 31IMRT	1996-2012	M 59.6 mo	*Toxicities:* Cyst growth: 19% vs 42% at 3 mo; 19% vs 32% after 3 mo Vascular morbidity: 10% vs 10% Vision: 5% vs 13% Hypothalamic obesity: 19% vs 29% Endocrinopathy: 76% vs 77% 3y OS: 94.1% vs 96.8% 3y CFFS: 67% vs 76.8% 3y NFFS: 91.7% vs 96.4%
(67) Sato et al. (2017)	Grade II-III pediatric intracranial ependymomas	PBT vs IMRT w/or wo/chemotherapy	PBT: M 55.8Gy (R 50.4-59.4Gy), IMRT: M 54Gy (R 50.4-59.4Gy) at 1.8Gy/fx in 28-33fx			41 PBT, 38 IMRT	2000-2013	PBT: M 2.6y (R 0.6-7.2y), IMRT: M 4.9y (R 1.1-11.7y)	3y PFS: 82% vs 60%* 3y OS: 97% vs 81%
(68) Yock et al. (2014)	Pediatric brain tumor	PBT vs photon therapy	< 50Gy or 50-54Gy or > 54Gy			57 PBT, 63 photon therapy	PBT: 2004-2009, photon therapy: 2001-2002	PBT: M 3y, photon therapy: M 2.9y	QoL: 75.9 vs 65.4*

Proton dose is RBE weighted.

*There is a statistically significant difference.

mo, month(s); y, year(s); fx, fraction(s); M, median; R, range; IQR, interquartile range; w, with; w/o, without; vs, versus; 3D-CRT, 3-D conformal radiation therapy; IMRT, intensity modulated radiotherapy; PBT, proton beam therapy; PSPT, passively scattering proton therapy; DSPT, double-scattering proton therapy; IMPT, intensity modulated proton therapy;

PBSPT, pencil beam scanning proton therapy; CNS, central nervous system; OS, overall survival; RFS, recurrence-free survival; CFFS, cystic failure-free survival; NFFS, nodular failure-free survival; GI, gastrointestinal; RBC, red blood cells; WBC, white blood cells; Hb, hemoglobin; CSI, craniospinal irradiation; IQ, intelligence quotient; FSIQ, full-scale intelligence quotient;

GAI, general ability index; VIQ, verbal reasoning; PIQ, perceptual reasoning; PSI, processing speed index; GAC, general adaptive composite; CM, cavernous malformations; ALC, absolute lymphocyte count; PLT, platelet count; PFS, progression-free survival; SMN, Secondary Malignant Neoplasms; QoL, quality of life; SIOP, International Society of Pediatric Oncology;

POG, Pediatric Oncology Group; CTCAE, Common Terminology Criteria for Adverse Events.

**Table 8:** Randomized clinical studies of proton versus photon radiation therapy.

Study	Tumor Type	Interventions	Dose	Number of patients	Accrual Period	Follow up	Clinical Outcomes (Proton therapy vs Photon therapy)
(69) Shipley et al. (1995)	Stage T3-T4Nx,0-2M0 prostate cancer	Photon boost therapy vs Proton boost therapy w/o ADT	Photon therapy: 50.4 Gy, 1.8 Gy daily, 5 fx/wk 16.8 Gy photons boost (total 67.2 Gy), 2.1 Gy daily, 4 fx/wk 25.2 Gy protons boost (total 75.6 Gy), 2.1 Gy daily, 4 fx/wk	93 proton boost therapy, 96 photon boost therapy	1982-1992	Proton boost therapy: M 62.1 mo (R 3-139 mo) Photon boost therapy: M 58.9 mo (R 5-138 mo)	*Toxicity:* Rectal bleeding: 32% vs 12% at 8y* Urethral stricture: 19% vs 8% at 8 y Hematuria: 14% vs 8% at 8y Urinary incontinence: 1% vs 1% at 8y Loss of full potency: 60% vs 63% Local tumor persistence/palpable and/or symptomatic regrowth: 6% vs 60%* DSS: 86% vs 83% at 5y, 67% vs 62% at 8y TRFS: 39% vs 41% at 5y, 20% vs 16% at 8y OS: 75% vs 80% at 5y, 55% vs 51% at 8y Local control: 86% vs 81% at 5y, 73% vs 59% at 8y
(70) Liao et al. (2018)	stage II-IV NSCLC	PSPT vs IMRT concurrent chemotherapy	74 or 66 Gy	57 PSPT, 92 IMRT	2009-2014	PSPT: M 25.7 mo (all patients), 48.8 mo (surviving patients) IMRT: M 24.1 mo (all patients), 36.4 mo (surviving patients)	Grade ≥ 3 pneumonitis: 10.5% vs 6.5% at 1y Local failure: 10.5% vs 10.9% at 1y
(71) Palma et al. (2019)	stage II-IV NSCLC	PSPT vs IMRT concurrent chemotherapy	66 or 74 Gy in 33 or 37 daily fx	64 PSPT, 114 IMRT	2009-2014	not specified	Pneumonitis of any grade: 36% vs 28% Symptomatic pneumonitis: 28% vs 19%
(72) Nantavithya et al. (2018)	stage I or recurrent NSCLC	SBPT (by using PSPT) vs SBRT (by using 3D-CRT/IMRT)	50 Gy in 4 12.5-Gy fx	10 SBPT, 9 SBRT	2012-2014	SBPT: M 36.5 mo SRBT: M 27 mo	3y OS: 90% vs 27.8% 3y PFSR: 70% vs 11.1% 3y local control: 80% vs 47.6% Tumor recurrence: 30% vs 66.7%
(73) Lin et al. (2020)	Stage I-III esophageal cancer	PSPT/IMPT vs IMRT concurrent chemotherapy	7 patients < 50.4 Gy (R 41.4-48.7 Gy), others 50.4 Gy in 28 daily fx	46 PBT (37 PSPT, 9 IMPT), 61 IMRT	2012-2019	M 44.1 mo	Posterior mean TTB: 17.4 vs 39.9* Mean POC score: 2.5 vs 19.1* 3y PFS: 44.5% vs 44.5% 3y OS: 50.8% vs 51.2% *QoL:* EQ-5D-5L: 0.81 ± 0.13 vs 0.83 ± 0.12 during treatment; 0.76 ± 0.15 vs 0.8 ± 0.14 at 1 mo 0.78 ± 0.15 vs 0.85 ± 0.13 at 3 mo 0.87 ± 0.12 vs 0.89 ± 0.12 after 3 mo VAS: 70.9 ± 15.9 vs 71.1 ± 20.2 during treatment 65.4 ± 18.9 vs 66.7 ± 20.8 at 1 mo 73.8 ± 17.6 vs 75.6 ± 20.0 at 3 mo 84.3 ± 12.8 vs 84.1 ± 16.7 after 3 mo
(74) Mohan et al. (2021)	Glioblastoma	PBT (PSPT/IMPT) vs photon therapy (IMRT/VMAT) concurrent chemotherapy	50 Gy and 60 Gy in 30 fx	28 PBT (20 IMPT, 5 PSPT, 3 IMPT + PSPT), 56 IMRT/VMAT	2014-2016	not specified	Grade ≥ 3 lymphopenia: 14% vs 39%*

Proton dose is RBE weighted.

*There is a statistically significant difference.

mo, month(s); wk, week(s); y, year(s); fx, fraction(s); M, median; R, range; w/o, without; vs, versus; ADT, androgen deprivation therapy; NSCLC, Non-small Cell Lung Cancer; IMRT, intensity modulated radiotherapy; PSPT, passively scattering proton therapy; 3D-CRT, 3-D conformal radiation therapy; IMPT, intensity modulated proton therapy; VMAT, volumetric modulated arc therapy; SBRT, stereotactic body radiation therapy; SBPT, stereotactic body proton therapy; PBT, proton beam therapy; DSS, disease-specific survival; TRFS, total recurrence-free survival; OS, overall survival; PFSR, progression-free survival rates; TTB, total toxicity burden; POC, postoperative complications; PFS, progression-free survival; QoL, quality of life;

EQ-5D-5L, European Quality of Life Five Dimension Five Level; VAS, visual analog scale.

**Table 9:** Non-randomized and randomized clinical trials of proton versus photon radiation therapy (see www.clinicaltrials.gov).

Registry Number	Study type	Status	Cancer type	Intervention	Primary Endpoint	Study Start Date	Estimated Primary Completion Date	Estimated Study Completion Date
Prostate cancer								
NCT04190446	Open-label, Randomized phase II study	Recruiting	Recurrent, Oligometastatic Prostate Cancer	Hypofractionated PBT vs Hypofractionated IMRT	The incidence of late (≥90 days post-treatment) grade ≥3 GI and/or GU adverse event	1/6/2020	12/31/2024	12/31/2024
NCT01617161	Open-label, Randomized phase III study	Recruiting	Low or Intermediate Risk Prostate Cancer	IMRT vs PBT	reduction in mean EPIC bowel scores at 24 months post-treatment	7/25/2012	12/1/2023	12/1/2026
NCT04083937	Single (Outcomes Assessor) blinded, Randomized phase III study	Recruiting	Prostate Cancer	Hypofractionated PBT vs Hypofractionated photon therapy	QoL	9/12/2018	4/1/2024	1/1/2028
NCT03561220	Open-label, Non-randomized study	Recruiting	Prostate Cancer	IMRT vs PBT	Bowel, urinary, and sexual dysfunction EPIC domain scores	7/5/2018	2/15/2026	4/1/2026
NCT02766686	Open-label, Non-randomized study	Recruiting	Prostate Cancer	IMRT vs PBT	Cumulative incidence of moderate/severe side effects	9/1/2016	8/1/2022	8/1/2023
Lung cancer								
NCT00915005	Open-label, Randomized phase II study	Completed	Locally Advanced Non-Small Cell Lung Carcinoma	Image-Guided Adaptive Conformal Photon Therapy vs PBT	The incidence and time to Development of CTCAE v3.0 Grade > 3 TRP; The incidence and time to development of local failure	6/1/2009	2/24/2020	2/24/2020
NCT01511081	Single (Participant) blinded, Randomized phase II study	Terminated (Low Accrual)	Centrally Located Stage I, Selected Stage II and Recurrent Non-Small Cell Lung Cancer	SBRT vs SBPT	Summary of 2-year grade ≥3 treatment-related toxicity	8/1/2012	10/1/2016	10/1/2016
NCT02731001	Open-label, Randomized study	Recruiting	Locally Advanced Non-small-cell Lung Cancer	IMRT vs PBT	Occurrence of acute and intermediate radiation induced side effects	8/1/2016	12/1/2021	12/1/2025
NCT01993810	Open-label, Randomized phase III study	Recruiting	stage II-IIIB non-small cell lung cancer	Photon therapy vs PBT	OS	2/3/2014	12/1/2024	12/31/2025
NCT01629498	Open-label, Randomized phase I/II study	Recruiting	Stage II-IIIB Non-small Cell Lung Cancer	Image Guided IMRT vs Image Guided IMPT	Survival free of grade ≥ 3 toxicity (with a target of at least 75%); LPFS (75% at 6 months)	9/17/2012	9/30/2022	9/30/2023
Breast cancer								
NCT04443413	Single (Outcomes Assessor) blinded, Randomized phase II study	Recruiting	Breast Cancer	Photon therapy vs PBT	Complication rate	6/8/2020	6/1/2022	6/1/2023
NCT04291378	Open-label, Randomized phase III study	Recruiting	Early Breast Cancer	Photon therapy vs PBT	Radiation associated ischaemic and valvular heart disease	6/1/2020	6/1/2027	6/1/2037
NCT02603341	Open-label, Randomized study	Recruiting	Non-Metastatic Breast Cancer	Photon therapy vs PBT	Effectiveness in reducing MCE, defined as atherosclerotic coronary heart disease or other heart disease death, myocardial infarction, coronary revascularization, or hospitalization for major cardiovascular event (heart failure, valvular disease, arrhythmia, or unstable angina)	2/1/2016	8/1/2022	11/1/2032
Esophageal cancer								
NCT03801876	Open-label, Randomized phase III study	Recruiting	Stage I-IVA Esophageal cancer	IMRT vs PBT	OS; Incidence of specific grade ≥ 3 treatment-induced cardiopulmonary AEs	3/15/2019	2/1/2027	2/1/2032
NCT01512589	Open-label, Randomized phase IIB study	Active, not recruiting	Esophageal Cancer	IMRT vs PBT	PFS; TTB	4/1/2012	4/30/2022	4/30/2023
NCT05055648	Open-label, Non-blinded, International Multicenter, Randomized phase III study	Not yet recruiting	Locally Advanced Esophageal Cancer	Photon therapy vs PBT	Pulmonary complications	10/1/2021	10/1/2024	10/1/2029
NCT03234842	Open-label, Non-randomized phase II study	Withdrawn (non-accrual)	Resectable and Unresectable Esophageal Cancer	IMRT vs PBT	Compare the rate of a clinically significant reduction of DLCO after preoperative or definitive chemoradiation	10/30/2017	12/11/2018	12/11/2018
Head and neck cancer								
NCT02923570	Open-label, Randomized phase II study	Recruiting	Head-and-Neck Cancer	IMRT vs PBT	Number of patients with grade ≥ 2 acute mucositis	10/3/2016	10/1/2022	10/1/2022
NCT03829033	Open-label, Randomized study	Recruiting	Early Tonsil Cancer	Photon therapy vs PBT	Acute and late side effects	1/22/2019	1/1/2028	1/1/2028
NCT01586767	Open-label, Non-randomized phase II study	Recruiting	Locally Advanced Sinonasal Malignancy	IMRT vs PBT	Local control rates	7/1/2011	7/1/2021	7/1/2024
NCT04607694	Open-label, Randomized study	Recruiting	Squamous Cell Carcinoma of the Pharynx or Larynx	Photon therapy vs PBT	Dysphagia ≥ grade 2; Xerostomia = grade 4	10/9/2020	3/9/2025	9/9/2025
NCT04528394	Open-label, Randomized phase II study	Recruiting	Nasopharyngeal Carcinoma	Photon therapy plus Carbon Ion Boost vs PBT plus Carbon Ion Boost	Number of participants with treatment-related xerostomia (≥ Grade 2)	4/29/2019	6/30/2021	6/30/2022
NCT01893307	Open-label, Randomized phase II/III study	Recruiting	Oropharyngeal Cancer	IMRT vs IMPT	Rates and severity of late grade 3-5 toxicity	8/26/2013	8/26/2023	8/26/2024
NCT04343573	Open-label, Multicenter, Randomized phase II study	Recruiting	Leptomeningeal Metastases	Proton CSI vs Involved-field Photon Radiation Therapy	CNS PFS	4/10/2020	4/1/2023	4/1/2023
Adult CNS cancer								
NCT01854554	Open-label, Randomized phase II study	Completed	Glioblastoma	IMRT vs IMPT	Time to Cognitive Failure	5/17/2013	10/13/2021	10/13/2021
NCT04752280	Open-label, Randomized study	Recruiting	Glioblastoma	IMRT vs PBT	Cumulative rate of toxicity	4/19/2021	8/19/2025	10/19/2027
NCT03180502	Open-label, Randomized phase II study	Recruiting	IDH mutant grade II or III glioma	IMRT vs PBT	Change in CTB COMP cognition score	8/2/2017	1/1/2025	1/1/2030
NCT04278118	Open-label, Non-randomized phase II study	Recruiting	Benign Intracranial Brain Tumors	Hypofractionated Photon Therapy vs Hypofractionated PBT	Local tumor control; Incidence of adverse events	2/18/2020	4/30/2023	4/30/2023
NCT02824731	Open-label, Non-randomized phase II study	Recruiting	Brain Tumors	Photon therapy vs PBT	Late toxicity	7/1/2016	7/1/2025	7/1/2026
NCT02179086	Open-label, Randomized phase II study	Not yet recruiting	Glioblastoma	Standard-dose 3D-CRT/IMRT vs Hypofractionated Dose-Escalated PBT	OS	10/27/2014	5/1/2024	5/1/2026
NCT04536649	Open-label, Multicenter, Randomized phase III study	Not yet recruiting	Glioblastoma	Photon therapy vs PBT and PBT plus Carbon Ion Boost	OS	10/1/2020	9/30/2023	9/30/2025
Other cancers								
NCT03186898	Open-label, Randomized phase III study	Recruiting	Unresectable or Locally Recurrent Hepatocellular Carcinoma	Photon therapy vs PBT	OS	6/22/2017	6/30/2024	6/30/2029
NCT04525989	Open-label, Randomized phase II study	Recruiting	Locally advanced rectal cancer	Photon therapy vs PBT	Incidence of acute grade 2-5 GI toxicity	4/20/2021	3/1/2028	3/1/2028
NCT04462042	Open label, Multi-center, Randomized phase II study	Recruiting	Anal Squamous Cell Carcinoma	VMAT/IMRT/Helical Tomotherapy vs IMPT	Acute grade >2 hematological side effects	4/7/2021	4/1/2025	3/28/2030
NCT04567771	Open-label, Early phase I study	Recruiting	Endometrial or Cervical Cancer	IMRT vs PBT	Change in EPIC bowel score	12/4/2020	10/15/2023	10/15/2024
NCT01659203	Open-label, Non-randomized phase I/II study	Recruiting	Retroperitoneal Sarcomas	Image Guided IMRT vs Image Guided IMPT	Local control rate	12/1/2012	8/1/2025	8/1/2025

3D-CRT, 3-D conformal radiation therapy; IMRT, intensity modulated radiotherapy; SBRT, stereotactic body radiation therapy; PBT, proton beam therapy; IMPT, intensity modulated proton therapy; SBPT, stereotactic body proton therapy; VMAT, volumetric modulated arc therapy; GI, gastrointestinal; GU, genitourinary; QoL, quality of life; EPIC, expanded prostate cancer index composite; CTCAE v3.0, common terminology criteria for adverse events, Version 3.0; TRP, treatment-related pneumonitis; OS, overall survival; LPFS, local progression-free survival; AEs, adverse events; PFS, progression-free survival; TTB, total toxicity burden; DLCO, diffusion lung capacity of carbon monoxide; MCE, major cardiovascular events; CSI, craniospinal irradiation; CNS, central nervous system; CTB COMP, Clinical Trial Battery Composite.

For endocrine metabolism following CSI, Bielamowicz et al. (56) showed that there was no statistically significant difference in primary and central hypothyroidism incidence between PSPT CSI and 3D-CRT CSI with IMRT boost in standard and high risk pediatric medulloblastoma patients in a single institutional study. However, an extension of this study with longer follow up time (median 5.6 years post-treatment) indicated that PSPT CSI treated patients had a lower risk of primary hypothyroidism than the patients in 3D-CRT CSI with IMRT boost group (57). No statistically significant differences in the risk of central hypothyroidism, growth hormone deficiency and adrenal insufficiency were found between groups. A propensity-matched multi-institutional study also reported that PSPT CSI resulted in a lower hypothyroidism rate, sex hormone deficiency incidence, and endocrine replacement therapy requirement than 3D-CRT/IMRT CSI for standard risk pediatric medulloblastoma patients after median 5.8 years post-treatment (58). There were no statistically significant differences in the risk of growth hormone deficiency, adrenal insufficiency, and precocious puberty between cohorts (**Table 7**).

For radiation-induced hematologic toxicity following CSI, a recently published multi-institutional retrospective study showed that DSPT CSI was associated with reduced acute hematologic toxicity, including leukopenia, lymphopenia, and anemia, as compared to photon CSI for a multivariable analysis in pediatric medulloblastoma patients, whereas the 5-year OS did not differ between the cohorts (59). Without adjusting for potential confounders, Song et al. (60) found there were lower incidences of grade ≥ 3 thrombocytopenia, platelet transfusion, and diarrhea in the proton CSI group than the photon CSI group for pediatric brain tumors (mainly medulloblastoma) patients at the National Cancer Center. Proton CSI was also associated with less reduction of white blood cells and platelets than photon CSI at one month post-treatment. In a single institutional study, Yoo et al. (61) also found PBSPT CSI patients had a lower decline of lymphocyte and platelet counts and lower risk of acute grade 3 anemia compared to 3D-CRT/helical tomotherapy CSI patients with pediatric brain cancer. However, there were no statistically significant differences in hemoglobin level, grade 3 thrombocytopenia, grade 4 lymphopenia, platelet transfusion, diarrhea and 3-year OS between groups without adjusting for potential confounders. Paulino et al. (62) also reported no statistically significant differences in 5- and 10-year OS and secondary malignant neoplasms risk between PSPT CSI and 3D-CRT CSI with IMRT boost for pediatric medulloblastoma patients. A multi-institutional case-matched study also showed that there were no significant differences in 6-year OS, 6-year recurrence-free survival and the patterns of failure between proton CSI and 3D-CRT/IMRT CSI in pediatric standard risk medulloblastoma patients (63) (**Table 7**).

In addition, for pediatric medulloblastoma patients, studies indicated that there were no statistically significant differences in grade 3 and 4 ototoxicity between PSPT CSI and 3D-CRT CSI with IMRT boost based on multiple evaluation scales (64), and cavernous malformations (CM) or CM-like lesions between proton and photon radiotherapy (65) (**Table 7**).

For cranial irradiation, a multi-institutional study of pediatric craniopharyngioma reported no statistically significant differences in 3-year OS, 3-year nodular failure-free survival and 3-year cystic failure-free survival between IMRT and proton radiation therapy (86% PSPT) using multivariable analysis (66). Based on the same database, Sato et al. (67) confirmed there was no statistically significant difference in 3-year OS between proton radiation therapy and IMRT for grade II-III pediatric intracranial ependymomas. However, proton radiation therapy was associated with higher 3-year progression-free survival compared to IMRT without adjusting for potential confounders. Nevertheless, another multi-institutional parent proxy-reported quality of life study showed that proton treated patients received better health related QoL than photon treated patients with pediatric brain tumor without adjusting for potential confounders (68) (**Table 7**).

To date, no randomized comparative clinical trial has been performed due to ethical barriers, since it would be difficult to suggest that there is clinical equipoise given the relative superiority of the proton dose distributions in such cases.

## 4 Discussion

According to the limited published comparative clinical studies mentioned above, the clinical benefit of proton radiation therapy is likely to vary between different radiation therapy techniques for different cancer sites, which makes it more difficult to demonstrate definitive advantages for proton radiation therapy.

Several esophageal cancer studies presented in this review have shown reduced incidence of radiation-induced toxicities (pulmonary, GI or cardiac toxicities) between PSPT and 3D-CRT, but no significant difference between PSPT and IMRT. This is likely because the highly conformal dose delivery capabilities in advanced photon therapy techniques (76, 77) may result in better clinical outcomes than 3D-CRT. Furthermore, there are no significant differences for radiation-induced GU/urinary toxicities in prostate cancer patients and for radiation-induced esophagitis and pneumonitis among NSCLC patients between proton therapy (mainly PSPT/DSPT) and IMRT. Therefore, proton therapy might not result in better clinical outcomes than intensity modulated photon treatment for certain cancer sites, for several potential reasons. First, the dose to organs at risk (OARs) can be maintained within tolerance doses using intensity modulated photon therapy for many of these sites. Therefore, the significant dose sparing capabilities of proton therapy may not translate into a remarkable clinical benefit. Second, a lack of significant clinical benefit in proton therapy may be due to the anatomic non-coincidence of the dose spared-regions and the regions that experienced radiation-induced toxicities. Palma et al. (71) presented that the significantly spared regions by PSPT as compared with IMRT are largely within the lower part of the lungs and the heart, whereas the radiation-induced pneumonitis affected regions are within the medial-anterior and upper parts of the thorax. Therefore, there was no statistically significant difference in radiation-induced pneumonitis between PSPT and IMRT for NSCLC patients. Cella et al. (23) also reported a similar occurrence in cardiac toxicity for NSCLC patients. The lack of superior effectiveness observed in the PSPT group may also be due to the lack of anatomic overlap between spared areas by PSPT as compared with IMRT and the areas that experienced radiation-induced pericardial effusion. Third, since proton beam delivery is very sensitive to tissue density changes, any anatomical variations or changes (e.g. patient weight, tumor changes, patient setup variation), and motions (e.g. respiration, cardiac activity, bladder filling) that occur in the beam path can have a much greater impact on the spatial dose distribution, resulting in substantially increased doses to OARs or reduced target dose coverage. To mitigate these anatomic changes and motions, development and application of robust optimization methods (78), tracking/gating techniques or breath hold methods (79, 80), in-room/real time image guidance (e.g. 4D-CT, cone-beam CT, MRI, optical surface monitoring system (OSMS)) (81) and *in vivo* range verification (e.g. positron emission tomography (PET), prompt gammas (PG) imaging) (82, 83) are currently being investigated. Fourth, uncertainty in the range of proton beams, due to uncertainties in CT data and the subsequent conversion to proton stopping power, results in substantial uncertainties in the delivered dose distribution. Dual-energy CT (DECT)-based SPR prediction (84) and proton CT (pCT) (85) may help overcome the limitation of CT Hounsfield unit-based SPR prediction (86) and thus potentially reduce the range uncertainty from 3.5% to 1.7% - 2.2% in DECT and 0.5% in pCT (87, 88). However, many aspects of these technologies are still in research and development. Last, the proton beam delivery techniques used in these studies are mainly PSPT or DSPT, which is a scattering process and generates a conformal dose at the distal side of the target volume, while the proximal side exhibits a much less conformal dose distribution. Modern proton techniques, such as PBSPT or IMPT, utilize a scanning rather than a scattering system to deliver uniform dose to a target volume in layers of proton “spots”, essentially “painting” the target volume (89). More comparative clinical studies between photon radiation therapy and these advanced proton techniques are needed to truly evaluate the effectiveness of proton radiation therapy in these cancer sites. Unfortunately, the clinical benefits of reducing the radiation-induced toxicities for breast cancer and adult CNS cancer are uncertain due to the limited amount of comparative clinical data.

Studies of head and neck cancers mainly focus on the comparison between PBSPT/IMPT and IMRT, and indicate that PBSPT/IMPT resulted in reduced gastrostomy tube usage and late radiation-induced xerostomia compared to IMRT. This benefit of proton therapy is likely due to 1) use of advanced proton spot scanning techniques and 2) the differences in beam delivery patterns and exit dose between proton and photon therapy, specifically that fewer proton beam entry paths and the lack of exit dose can potentially avoid many critical structures. These advantages of proton therapy also translate into a superior clinical benefit among pediatric CNS cancer patients. The studies showed that craniospinal proton radiation therapy (PSPT/IMPT) is most likely associated with reduced short-term (< 5 years post-treatment) effects on pediatric patient cognitive development, long-term (median 5.7 years post-treatment) hypothyroidism incidence and hematologic toxicities as compared to craniospinal photon radiation therapy (3D-CRT/IMRT). Interestingly, these comparative studies also indicated that proton radiation therapy (mainly PBSPT/IMPT) is most likely associated with a lower severe radiation-induced lymphopenia rate compared to IMRT in thoracic and craniospinal radiation therapy. T-lymphocytes play a central role in anticancer immune response and severe treatment-related lymphopenia is associated with poor survival rates in chemotherapy and/or radiotherapy (90, 91). Cho et al. (92) demonstrated that radiation treatment-related lymphopenia is also correlated with inferior survival rates for NSCLC patients treated with immunotherapy. The lymphocyte sparing achieved from advanced radiation techniques is certainly beneficial, however, whether it could enhance anticancer immune response and improve survival rates remains to be verified. While the studies in this review showed that the survival rates may not be significantly improved with proton radiation therapy for either adult or pediatric cancers, this is to be expected for studies in which the target dose is similar regardless of the delivery technique. Better dose shaping and reduction of uncertainties in dose calculation and delivery can reduce treatment margins, which can reduce normal tissue doses and potentially allow for dose escalation in the target. An ongoing clinical trial (NCT02179086) may provide valuable evidence of improved survival by comparing dose-escalated proton therapy vs standard-dose photon therapy.

Such studies, however, have been met with challenges. Due to issues surrounding equipoise, treatment costs, insurance coverage, and the relatively small number of charged-particle radiation therapy centers in operation, it can be difficult to activate and complete a randomized controlled clinical study in a timely manner. Therefore, most of the currently available data are non-randomized retrospective studies, which contain inevitable misclassification and selection biases despite adjustment for potential confounders by multivariable regression analysis and propensity/case-matched analysis. Moreover, the studies based on multi-institutional or medical databases potentially involve heterogeneity in treatment protocols and techniques. Some of the studies even use both older and modern delivery techniques, such as PSPT/DSPT and PBSPT/IMPT techniques for proton radiation therapy, and 3D-CRT and IMRT techniques for photon radiation therapy, without presenting the number of patients treated with each technique. Some studies did not indicate the type of proton or photon techniques they used. This could cause an imprecise comparison between proton and photon therapies since PBSPT/IMPT can offer better healthy tissue sparing and may result in lower toxicities than PSPT/DSPT (93). Finally, small sample size, limited follow-up duration and preconceived bias from patient-reported outcomes could also increase the uncertainty of study conclusions.

## 5 Conclusion

This review has presented currently available comparative clinical outcomes between proton and photon therapies for several cancer types. Overall, passive scattering proton therapy shows similar clinical outcomes to intensity modulated photon therapy for prostate, lung and esophageal cancers, while active scanning proton therapy appears to result in a decrease in certain radiation-induced side effects as compared to intensity modulated photon therapy for head and neck, thoracic, craniospinal, and pediatric CNS cancers. However, the evidence is not definitive and further demonstration of the clinical benefit of proton radiation therapy will depend on the findings of ongoing and future comparative randomized clinical trials. In the meantime, further development of beam delivery and imaging techniques is necessary to fully take advantage of the dose shaping capabilities of proton radiation therapy and achieve its full clinical potential.

## Author contributions

ZC and JB contributed to the scope and design of the review. ZC performed the resources search and original draft. ZC, JB, MD, MJ revised and edited the draft. All authors contributed to the manuscript and approved the submitted version.
